# Multiple Object Tracking for Dense Pedestrians by Markov Random Field Model with Improvement on Potentials

**DOI:** 10.3390/s20030628

**Published:** 2020-01-22

**Authors:** Peixin Liu, Xiaofeng Li, Yang Wang, Zhizhong Fu

**Affiliations:** School of Information and Communication Engineering, University of Electronic Science and Technology of China (UESTC), 2006 Xiyuan Avenue, Chengdu 611731, China; pxl@std.uestc.edu.cn (P.L.); wyang@std.uestc.edu.cn (Y.W.); fuzz@uestc.edu.cn (Z.F.)

**Keywords:** multi-camera multi-object tracking, dense pedestrian crowds, cross-view data fusion, image mutual information, Markov random field model

## Abstract

Pedestrian tracking in dense crowds is a challenging task, even when using a multi-camera system. In this paper, a new Markov random field (MRF) model is proposed for the association of tracklet couplings. Equipped with a new potential function improvement method, this model can associate the small tracklet coupling segments caused by dense pedestrian crowds. The tracklet couplings in this paper are obtained through a data fusion method based on image mutual information. This method calculates the spatial relationships of tracklet pairs by integrating position and motion information, and adopts the human key point detection method for correction of the position data of incomplete and deviated detections in dense crowds. The MRF potential function improvement method for dense pedestrian scenes includes assimilation and extension processing, as well as a message selective belief propagation algorithm. The former enhances the information of the fragmented tracklets by means of a soft link with longer tracklets and expands through sharing to improve the potentials of the adjacent nodes, whereas the latter uses a message selection rule to prevent unreliable messages of fragmented tracklet couplings from being spread throughout the MRF network. With the help of the iterative belief propagation algorithm, the potentials of the model are improved to achieve valid association of the tracklet coupling fragments, such that dense pedestrians can be tracked more robustly. Modular experiments and system-level experiments are conducted using the PETS2009 experimental data set, where the experimental results reveal that the proposed method has superior tracking performance.

## 1. Introduction

Video multiple object tracking (MOT) is widely used in computer vision research applications, including video surveillance, traffic detection, and robotic assistance. With developments [1,2,3,4,5] in object detection technology, tracking by detection (TBD), such as in [6,7,8,9], has become a common tracking strategy. This tracking scheme performs data association based on the appearance and motion characteristics of detected information to obtain the complete trajectories of objects.

**Figure 1:**
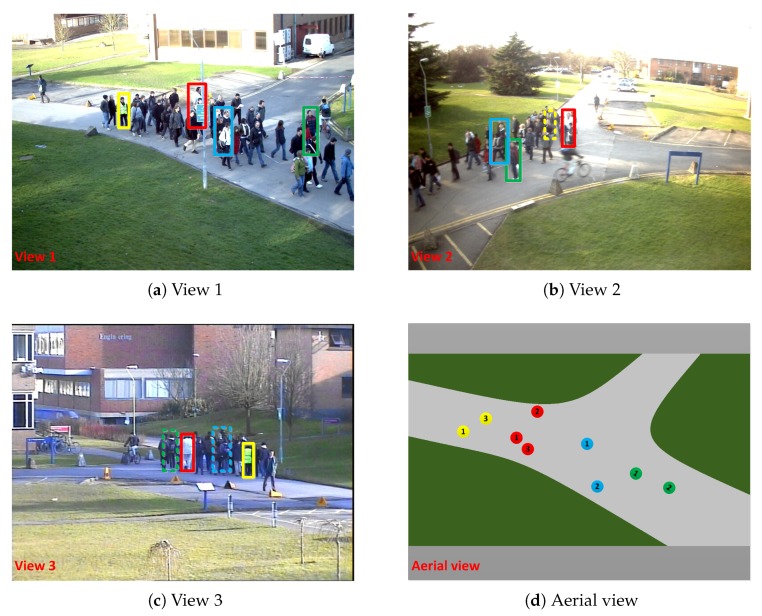
Occlusions in a multi-camera system. (**a**–**c**) Images of the 233rd frame from the first, second, and third views in PETS2009 S2.L3, respectively. These three images jointly illustrate the severe occlusions caused by dense crowds and the consequent deviations in detection. The solid-line bounding box is the detection result, while the dotted-line bounding box is the expected result of the occluded objects. (**d**) The positions of objects in aerial view produced by multi-view 3D reconstruction. Due to the presence of detection noise, the multi-view reconstruction results of each object do not coincide exactly.

One of the major challenges in TBD is object occlusion. In a single-camera scene, trajectory estimation and data association [10,11] are used to deal with occlusion; however, frequent long-term occlusions cased by dense pedestrians scene may have a large effect and result in a significant reduction in tracking performance. In a multi-camera system with overlapping fields of view, some kinds of occlusions can be effectively solved by cross-view data fusion [12,13,14,15]. Figure 1a–c presents the first, second, and third view video frame images, respectively, of the 233rd frame of the PETS2009 experimental data set S2.L3. The man in yellow vest (displayed in the dashed yellow bounding box) is occluded by the crowd in the second view; however, he is visible in the first and third views (solid yellow bounding box). By data fusion of the first and third views, this kind of occlusion problem can be effectively solved.

However, one of the most difficult challenges for multi-camera systems [12,13,14] is the tracking of dense pedestrians, in which several views or all views are largely or partially occluded. As illustrated in Figure 1, the object represented by the blue bounding box is partially occluded in Figure 1a and completely occluded in Figure 1c; similarly, the objects represented by red, yellow, and green are also occluded to varying degrees. These occlusions lead the target detection information to be highly inaccurate, resulting in large errors in the 3D reconstruction information of the same target from different cameras (as illustrated in Figure 1d), which can lead to errors in multi-view data fusion. In addition, due to the group motion characteristics of dense pedestrians, the complete occlusion points typically change with changes in time and position, which may result in a large number of short tracklets when the trajectory fragments are established. With insufficient information, these short tracklets cannot provide reliable features of the objects, thus resulting in a decline in data association performance. The two above-mentioned problems caused by dense object occlusions both have a large impact on multi-view multiple object tracking performance.

In this paper, the multi-camera tracking system builds cross-view tracklet couplings with a new data fusion method, and links them by association algorithm based on a new Markov random field (MRF) model. To address the problem of inaccurate detection caused by frequent occlusions in dense crowds, the human key points detection method [5] is used to improve the object positions. Then, two-dimensional (2D) tracklets are generated in each view and are reconstructed in three dimensions using camera parameters. A proposed data fusion method is used to calculate the spatial similarity of the cross-view tracklets, based on image mutual information. This method takes into account the position and motion relationships between two tracklets.

The proposed MRF model uses the link candidates of two tracklet couplings as the observation node and their internal link state as the implicit node. The MRF model contains a new potential function improvement method for dense pedestrian crowd scenarios, including assimilation as well as extension processing and a message selective belief propagation (MSBP) algorithm. The former enhances the information of the short tracklet coupling and expands it through information sharing. The latter prevents the unreliable messages of short tracklet couplings from spreading throughout the network. The potentials of the model are improved with the help of iterative belief propagation processing [16,17]. Consequently, an effective association of the tracklet coupling fragments is achieved and improved tracking performance of dense pedestrian crowds obtained.

The main contributions of this paper are as follows.

(1) We propose a cross-view data fusion method based on image mutual information. Together with human key point optimization, it can generate more reliable tracklet couplings.
(2) An MRF model is constructed and the potential function improvement method is proposed for better association of short cross-view tracklet couplings.
(3) We construct a complete multi-view MOT system, which is tested on public data sets containing dense pedestrian scenarios and achieves favorable results.

The rest of this paper is organized as follows. Related works in Section 2; generation of cross-view tracklet coupling is described in Section 3; system framework and Markov random field model for data association are introduced in Section 4; experiments are shown in Section 5; Section 6 is the discussion and conclusion; the last section is the Appendix A.

## 2. Related Works

TBD is an effective solution in MOT, the main task of which is to determine the complete trajectories of objects through data association and estimation processing of the detected information. Multi-view MOT with overlapping fields of view has been designed to improve tracking performance by performing multi-view data fusion. However, a multi-view tracking system is more complex than a single-view system, and much research [18,19,20,21] has been conducted on this topic. In this section, we discuss multi-view data fusion and association.

Berclaz et al. [22] designed a probabilistic occupancy map [23] for multi-camera tracking systems to achieve the 3D reconstruction of multi-view detection information. They modeled data association as a linear programming problem and proposed the k-shortest paths algorithm. Dockstader et al. [18] constructed a complete multi-view MOT system and used a Bayesian belief network to accomplish multi-view data fusion. In addition, they adopted a Kalman filter to estimate the trajectories. For tracking in dense pedestrian crowds, Eshel et al. [19] established a multi-camera system with overlapping views. Placing the cameras at higher positions facilitated capturing the heads of objects, and robust tracking in a dense crowd was achieved based on head detection. In [15] and [20], thorough discussions of the reconstruction before tracking and tracking before reconstruction frameworks and proposed improvement measures were provided. Leal-Taixé et al. [12] proposed a global optimization scheme, which establishes a multi-layer graph model based on reconstruction matching and data association and solves it based on a multi-commodity flow algorithm. It takes the distance metric into consideration in 3D reconstruction and adopts the metric function proposed in [24] to convert the absolute distance into a probability. This function decreases gently within the threshold and declines rapidly when the distance is greater than the threshold, which may improve the performance of the system against detection noise. Hofmann et al. [13] established a multi-view network flow tracking model to simultaneously describe multi-view information reconstruction and time-domain data association. In addition, they added multi-view reconstruction to the network flow graph as an additional constraint. Wen et al. [14] constructed a global hypergraph model to describe multi-view reconstruction and tracking, taking into account high-order dependencies among nodes in addition to simple domain relationships. Duanmu et al. [25] proposed a multi-view MOT system, which generated tracklets in each view and used the graph matching method to solve the cross-view association problem. Nie et al. [26] proposed a general tracking framework for single-view and multi-view systems, which transformed the data association of tracklets into graph matching problems. Nithin et al. [27] proposed a grammar model with stochastic attributes to improve cross-view tracking performance using complementary and distinguishing attributes. In the association framework, Liu et al. [28] modeled the association of tracklets as a combination optimization problem based on appearance motion and geometric information, while considering the long-term and short-term occlusion problems and improving system efficiency.

In [13,14,25,26], Euclidean distance metrics have been used as cross-view metrics. Due to the ubiquitous detection noise caused by object occlusion, errors can occur during 3D reconstruction in single-view object detection. It is common to use the absolute distance directly to calculate the 3D reconstruction differences between detections at different views. However, this method is too sensitive to detection noise and may cause matching errors in difficult situations. Leal-Taixé et al. [12] considered this factor and used the Gaussian error function for calculation, which can improve the robustness of the matching. In this paper, we also considered the metric’s ability to suppress detection noise, set a reasonable error threshold, and used the image mutual information metric to provide the matching index.

In data association-based MOT research, an effective method is to use a probability graph model solution, which can be globally optimized and achieves favorable tracking performance. The network flow tracking model presented in [8] clearly describes the relationship between detections and plans a possible match for all objects as a whole. It can also simultaneously solve the complete trajectories of multiple objects. There are a number of variants [29,30,31] in which, for instance, nodes have different meanings and different methods are used to describe the relationships between nodes. Due to its clear structure, the network flow tracking model has become one of the most popular tracking models. To handle more complex tracking scenarios, conditional random field models have been widely used. Yang et al. [32] added a high-order trajectory continuity constraint to ensure the reliability of matching while paying attention to the node connections. To deal with object pairs with similar positions and appearances, the authors of [7] modeled the relationship between such pairs as the edge of the conditional random field and mapped this relationship as a data association problem, with the binary energy function as a constraint, to robustly solve problems in complex situations. Milan et al. [33] modeled the trajectory smoothness problem [9] in tracking as a unitary energy function and modeled mutual exclusion as a binary energy function. They performed optimization and achieved favorable results. In [34,35,36,37,38], deep learning technology has been further applied to the conditional random field tracking model, in order to improve the distinction degree of object features. In [6,11], a larger range of node relationships were considered and a hypergraph model was established to address the data association problem. However, among the many existing data association models, it is more likely that the reliability of the nodes and the weights on the edges will be trusted. In an object-intensive scene, due to the ubiquity of occlusion, tracklets are too short to provide sufficient information. This situation makes the relationships between tracklets unreliable, and consequently data associations based on these unreliable relationships can lead to the generation of a large number of erroneous trajectories. In this study, the Markov optimization model is established for the data association of cross-view tracklets and a potential function improvement method is proposed to increase the reliability of the relationships between the nodes, which lay the foundation for trajectory generation.

## 3. Generation of Cross-View Tracklet Coupling

In the tracking framework, a 2D tracklet set $T^{v}$ is generated for each view, based on the detection input (where v∈V represents the view). Tracklets in multiple views are sequentially merged by data fusion to generate a multi-view tracklet coupling set T. To reduce the influence of detection noise caused by occlusion on data fusion accuracy, the human key points detection method is used to optimize the object detection in each view, thereby reducing the 3D reconstruction error of the object. A cross-view tracklet measurement method based on image mutual information is proposed, which can more accurately describe the spatial relationship between the cross-view tracklets and obtain a multi-view fusion tracklet coupling set through an iterative generation algorithm.

### 3.1. Object Position Data Optimization Based on Human Key Points Detection

The basis of multi-view data fusion is the 3D reconstruction of objects; that is, mapping the 2D detection information of multiple views into a unified 3D space. Generally, only the object landing location (center position at the bottom of the 2D detection bounding box) is 3D mapped [14] to reconstruct the pedestrian object to the ground of the public 3D space, which can effectively reduce the computational complexity. In each view, the detection algorithm provides an approximate outline of the object. Most solutions provide a rectangular area [8,14] containing the object; however, some methods provide an elliptical area [32]. The detection bounding box is generally accurate under the condition that the object is not substantially occluded. In a typical 3D reconstruction process, it is assumed that the precalibrated camera parameters are accurate and that only accurate 2D detection data can reconstruct reliable 3D position. However, the detection contains some noise and, for an object, there is always a deviation between the center point of the target detection bounding box at the bottom of the same target and the corresponding real point in 3D. In this study, these two deviations are collectively referred to as detection errors. As the connection between the camera and the object’s landing location usually forms an acute angle (of less than 45 degrees) with the ground, the error caused by detection is magnified when mapped by the camera parameters into a 3D space. For dense crowd scenarios, as illustrated in Figure 1, a slightly severe occlusion causes large errors or even false detections of the object. Large errors occur when 3D position reconstruction is performed directly through the bottom center of the detection bounding box, which can lead to the failure of data fusion. Therefore, conducting multi-view data fusion based on existing detection methods is often not reliable. This study makes use of the human key points detection method to optimize detection information to obtain a more accurate object 3D reconstruction position, reduce the reconstruction error, and lay a solid foundation for multi-view data fusion.

**Figure 2:**
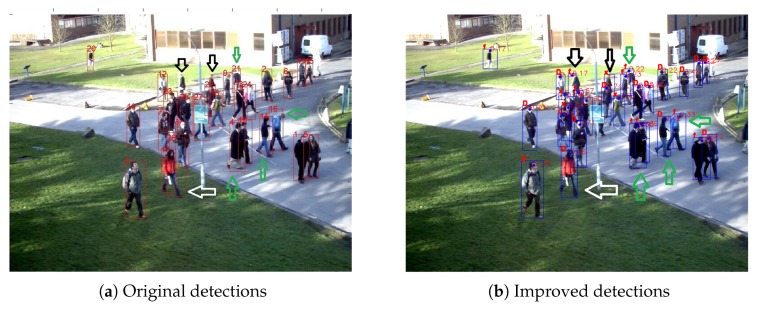
Results of the optimization method based on human key points detection. (**a**) The original detections; (**b**) the optimized results. The white arrow indicates the processing of a false detection, the black arrow indicates the compensation of missed detection using the key points, and the green arrow indicates that key point information is used to optimize not only the coordinates of the bottom points, but also the overall detection bounding box.

We adopt the human key point detection algorithm proposed in [5]. For the detection $d_{i}^{t}$ whose confidence is less than a threshold $\delta_{c}$, the human key point detection is executed on the area slightly larger than the detection. If detection overlapping happens due to dense objects, the processing area will be doubled. The obtained key point data $KP(d_{i}^{t})$ is used to optimize the original detection. Detection optimization based on key point information can accomplish three tasks—removing error detection, finding missed detection, and correcting the detection bounding box—as shown in Figure 2 by white, black, and green arrows respectively. The optimization is mainly based on head key point $KP_{h}\left(d_{i}^{t}\right)$ which is often available. If not severe occluded, as the 4th, 14th, and 16th detections indicated by the green arrows in Figure 2, the foot key point $KP_{f}\left(d_{i}^{t}\right)$ can also be obtained. The top and bottom of the bounding box is then refined by $KP_{h}\left(d_{i}^{t}\right)$ and $KP_{f}\left(d_{i}^{t}\right)$. Due to the uncertainty of the pedestrian’s posture, prior knowledge is used for width of detections instead of shoulder key points. If part of key point information is unavailable, prior knowledge will be used. When the output of human key points detection algorithm is completely null, the detection is taken as false and deleted, as the $d_{2}^{t}6$ at the white arrow in Figure 2a. In addition, the processing area is an extension of the original detection area $d_{i}^{t}$. If $d_{i}^{t}$ is in a dense crowd, the processing area will contain other pedestrians, that is, $KP(d_{i}^{t})$ contains multiple groups of key point information. This is helpful for missing objects. For example, the pedestrians at black arrows were not detected in Figure 2a and is now found by human key point detection algorithm in Figure 2b. In a densely crowded area, processing areas may overlap, resulting in multiple detection of an object. Therefore, overlapping processing is required.

### 3.2. Cross-View Tracklet Spatial Relationship Metric Based on Image Mutual Information

If object detections $d_{i}^{t}\left(m\right)$ and $d_{j}^{t}\left(n\right)$ from views *m* and *n* correspond to the same object, then the 3D coordinates of their landing locations should theoretically overlap. Due to the detection errors, the positions of their landing locations in the 3D reconstruction may become deviated, which may significantly affect the quality of cross-view image fusion in dense crowd situations.

Based on 2D tracklets, we perform data fusion from three dimensions in this study: time, space, and view. To achieve correct matching, it is necessary to accurately measure the spatial relationships between tracklets from different views. We take two 2D tracklets as an example, where $T_{i}^{m}$ is the *i*th tracklet of view *m* and $T_{j}^{n}$ is the *j*th tracklet of view *n*. To measure the spatial relationship between $T_{i}^{m}$ and $T_{j}^{n}$, the authors of [13,14] provide the calculation method for combined dispersion. In [12], a Gaussian metric was used to calculate the positional similarity between tracklets. Both of these schemes can tolerate the position errors in 3D object reconstruction. However, in addition to the positional relationship between $T_{i}^{m}$ and $T_{j}^{n}$, the motion relationship between them should also be taken into consideration. In studies of image registration [39,40], an effective method is to use mutual information as the similarity between two images *a* and *b*. Based on the reference image *b*, the geometric transformation of the input image *a* is carried out iteratively and the mutual information of *a* and *b* is calculated. When the mutual information reaches maximum, the optimal registration parameters are obtained. Inspired by this, we propose a spatial similarity measurement method that can comprehensively consider the position and motion information of two tracklets; that is, the method uses the image mutual information to calculate the spatial relationship between the tracklets.

**Figure 3:**
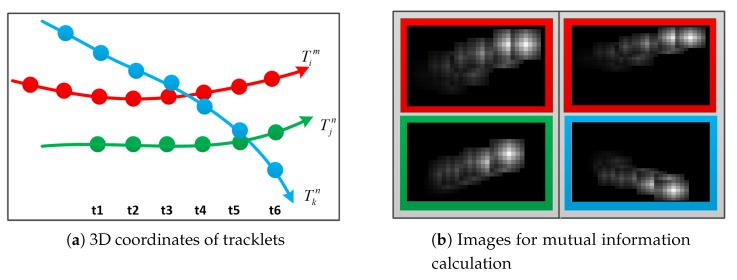
Calculation of tracklet spatial relationship based on image mutual information: (**a**) Illustration of the 3D trajectory projection z=0 of three tracklets. The red tracklet $T_{i}^{m}$ is from view *m*, while the blue and green tracklets, $T_{j}^{n}$ and $T_{k}^{n}$, are both from view *n*. The left images in panel (**b**) present grayscale images corresponding to the two tracklets in the calculation process of the mutual information $MI(T_{i}^{m},T_{j}^{n})$, while the images on the right are the images of $T_{i}^{m}$ and $T_{k}^{n}$.

As illustrated in Figure 3a, the 3D coordinates of $T_{i}^{m}$ and $T_{j}^{n}$ are first reconstructed using the calibrated camera parameters and detection information. Generally, it is assumed that, in a real tracking scene, the ground is flat and fluctuations can be neglected; furthermore, reconstruction is performed by utilizing only the center points at the bottom of the detection bounding boxes to obtain the 3D coordinates (z=0) of the object’s landing points. When considering the spatial relationship between $T_{i}^{m}$ and $T_{j}^{n}$, the distance between the detections corresponding to the same frame is usually calculated and summed as follows.

(1) $$\begin{array}{c} F\left(T_{i}^{m},T_{j}^{n}\right)=\sum_{t}D(T_{i}^{m}\left(t\right),T_{j}^{n}\left(t\right)). \end{array}$$

As the presence of detection noise causes each 3D reconstruction coordinate to deviate from the true value, this noise may affect the calculation of the positional relationship. The Gaussian metric can tolerate reconstruction errors within a certain range (σ) and attenuate distances outside this range. In this study, the image mutual information method can simultaneously satisfy the three requirements for the spatial similarity calculation for the tracklets; namely, positional relationship, noise tolerance, and motion relationship.

To calculate the spatial similarity between two tracklets, they are described as two 8-bit grayscale images ($I_{i}^{m}$ and $I_{j}^{n}$, respectively) with time-overlapping tracklets taken into consideration. As illustrated in Figure 3b, the background color is set to black; that is, the pixel value is set to 0. When $I_{i}^{m}$ or $I_{j}^{n}$ is established, a Gaussian gray block is constructed, frame-by-frame, with the reconstructed coordinates as the center. The scale of the gray block corresponds to the range of noise tolerance and the Gaussian attenuation reflects the weight attenuation of the deviation from the reconstructed coordinate point. The gray base values of each Gaussian block are incremented, step-by-step, in increasing order of $T_{i}^{m}$ and $T_{j}^{n}$ to indicate their direction of motion. The overlapping relationship between each Gaussian window can express the velocity information of the tracklets. By calculating the mutual information $MI(I_{i}^{m},I_{j}^{n})$ of the two images, the spatial similarity of $T_{i}^{m}$ and $T_{j}^{n}$ can be obtained.

(2) $$\begin{array}{c} MI\left(T_{i}^{m},T_{j}^{n}\right)=H\left(I_{i}^{m}\right)+H\left(I_{j}^{n}\right)-H\left(I_{i}^{m},I_{j}^{n}\right). \end{array}$$

An improved distinguishing effect can be achieved by using the image mutual information to calculate the spatial relationship between two tracklets. As illustrated in Figure 3a, it is assumed that $T_{i}^{m}$ and $T_{j}^{n}$ belong to the same object, whereas $T_{i}^{m}$ and $T_{k}^{n}$ belong to different objects. As the motion trajectories of $T_{i}^{m}$ and $T_{k}^{n}$ overlap, the distance superposition calculation or average distance calculation method would be unable to distinguish them effectively. However, the image mutual information can distinguish them well and, as seen in Figure 3b, there are significant differences between the images of $I_{i}^{m}$ and $I_{k}^{n}$.

As the lengths and distances between tracklets differ, there are differences in the sizes of each pair of images. Therefore, it is also necessary to standardize the mutual information to perform a unified comparison. To this end, we have set all images to the same size. Assuming that the information entropy of a certain image with a size of $N_{0}$ is $H_{0}\left(x\right)$, it can be easily proven that, when its size is expanded to $N_{0}+N_{a}$ by adding zero value pixels, its information entropy $H_{1}\left(x\right)$ satisfies the following,

(3) $$\begin{array}{c} H_{1}\left(x\right)=\beta H_{0}\left(x\right)+f\left(n_{0},N_{0},N_{a}\right), \end{array}$$

where $\beta =N_{0}/\left(N_{0}+N_{a}\right)$, $f(n_{0},N_{0},N_{a})$ is a function of $n_{0}$, $N_{0}$, and $N_{a}$, and $n_{0}$ is the number of zero value pixels in original image $N_{0}$. The specific form and the proof process of Equation (3) are given in the Appendix A.

### 3.3. Iterative Generation for Tracklet Couplings

In this section, an iterative generation algorithm for multi-view tracklet coupling is designed, based on the cross-view tracklet mutual information metric proposed in Section 3.2. According to Equation (4), the multi-view data fusion problem can be described as the problem of identifying a set of tracklet couplings that can obtain the maximum mutual information:

(4) $$\begin{array}{c} \begin{array}{c} T^{*}=\underset{T}{\arg\max}\sum_{i}MI(T_{i})\\ =\underset{T}{\arg\max}\sum_{j}\sum_{k}MI(T_{j}^{m},T_{k}^{n})\\ s.t.f_{t}\left(T_{j}^{m},T_{k}^{n}\right)=1,f_{a}\left(T_{j}^{m},T_{k}^{n}\right)=1, \end{array} \end{array}$$

where $T=\{T_{i}\}$ is the set of tracklet couplings; $T_{i}$ is the union of $T_{j}^{v}$, defined as $T_{i}=\underset{j,v}{⋃}T_{j}^{v}$; and $T_{i}\neq \emptyset$ contains at least one tracklet. $MI(T_{j}^{m},T_{k}^{n})$ is the spatial similarity between tracklet pair $\left(T_{j}^{m},T_{k}^{n}\right)$. $T_{j}^{m},T_{k}^{n}\in T_{i}$, m,n∈V and m≠n. In the cross-view fusion process, the 2D tracklets must meet the temporal overlap of $f_{t}\left(T_{j}^{m},T_{k}^{n}\right)=1$ and the appearance consistency of $f_{a}\left(T_{j}^{m},T_{k}^{n}\right)=1$ before they can be coupled.

(5) $$\begin{array}{c} f_{t}\left(T_{j}^{m},T_{k}^{n}\right)=\left\{\begin{array}{c} 1\;if\;t\left(T_{j}^{m}\right)\cap t\left(T_{k}^{n}\right)\neq \emptyset \\ 0\;else \end{array}\right. \end{array}$$

Using $T_{j}^{m}$ and $T_{k}^{n}$ as an example, $t(T_{j}^{m})$ is the frame list of $T_{j}^{m}$, while $t(T_{k}^{n})$ is the frame list $T_{k}^{n}$. If $T_{j}^{m}$ and $T_{k}^{n}$ can be coupled, then they contain at least one pair of detections that have the same frames; that is, there is a temporal overlap relationship, as presented in Equation (5).

Thus, $T_{j}^{m}$ and $T_{k}^{n}$ must meet cross-view appearance constraints at the same time, according to Equation (7). Due to the difference in views, there can be large differences in the colors of the cross-view images; furthermore, the difference in the angle of the field of view can cause the texture characteristics of the same object to be quite different in different views. These create challenges in the calculation of cross-view appearance similarity. An effective processing scheme is to use a neural network method for online training to extract the differentiated appearance features of the cross-view object. Due to the complexity of the online training process, it will be studied in follow-up work. In Equation (6), the current study applies a traditional color histogram to calculate the cross-view appearance constraint, using the Bhattacharyya function $B(h\left(T_{j}^{m}\left(p\right)\right),h\left(T_{k}^{n}\left(q\right)\right))$ for the similarity calculation function. Before the calculation, color deviation preprocessing is performed for multiple views to reduce the calculation error caused by color deviation.

(6) $$\begin{array}{c} \Lambda_{a}^{c}\left(T_{j}^{m},T_{k}^{n}\right)=\frac{1}{N}\sum_{p}\sum_{q}B(h\left(T_{j}^{m}\left(p\right)\right),h\left(T_{k}^{n}\left(q\right)\right)). \end{array}$$

$T_{j}^{m}$ and $T_{k}^{n}$ can perform cross-view coupling, and their appearance similarity $\Lambda_{a}^{c}\left(T_{j}^{m},T_{k}^{n}\right)$ must be greater than the set threshold $\delta_{a}^{c}$, as indicated in Equation (7). Correspondingly, $\delta_{a}^{i}$ is the threshold for the same-view calculation.

(7) $$\begin{array}{c} f_{a}\left(T_{j}^{m},T_{k}^{n}\right)=\left\{\begin{array}{c} 1\;if\;\Lambda_{a}^{c}\left(T_{j}^{m},T_{k}^{n}\right)\geq \delta_{a}^{c}\;\&\;m\neq n\\ 1\;if\;\Lambda_{a}^{i}\left(T_{j}^{m},T_{k}^{n}\right)\geq \delta_{a}^{i}\;\&\;m=n\\ 0\;else. \end{array}\right. \end{array}$$

In the process of coupling, as illustrated in Algorithm 1, if $T_{i}$ already contains other 2D tracklets $T_{l}^{n}$ in view *n*, then $T_{k}^{n}$ must also satisfy the appearance constraints of $T_{l}^{n}$ from the same view before the coupling, as demonstrated in Equation (7), in which the calculation of appearance similarity in the same view also adopts the traditional color histogram method, as shown in Equation (8).

(8) $$\begin{array}{c} \Lambda_{a}^{i}\left(T_{k}^{n},T_{l}^{n}\right)=B\left(h_{c}\left(T_{k}^{n}\right),h_{c}\left(T_{l}^{n}\right)\right). \end{array}$$

**Algorithm 1** Tracklet coupling iterative generation algorithm**Input:** 2D tracklet information for each view**Output:** Tracklet coupling set $T=\{T_{i}\}$ 1:Set T=∅.2:Calculate the spatial similarities between all tracklets, according to Equation (2).3:Arrange those that exceed the threshold (total number N) in descending order of association strength.4:**while** There exist incomplete processed tracklet pairs **do**5:    Find the max and incomplete processed pair $\left(T_{j}^{m},T_{k}^{n}\right)$.6:    **if** the current $\left(T_{j}^{m},T_{k}^{n}\right)$ does not belong to any existing $T{}_{i}$ **then**7:        Form this $\left(T_{j}^{m},T_{k}^{n}\right)$ into new $T_{i+1}$, $T_{i+1}=\left\{T_{j}^{m},T_{k}^{n}\right\}$.8:    **else if** the current $T_{j}^{m}$ only belongs to one existing $T{}_{i}$ **then**9:        **if** $T_{k}^{n}$ and $T{}_{i}$ satisfy the Equation (5) and (7) **then**10:           Update $T{}_{i}=\left\{T_{j}^{m},T_{k}^{n}\right\}$.11:        **else**12:           **if** $\left(T_{j}^{m},T_{k}^{n}\right)$ has been unprocessed **then**13:               Mark the pair $\left(T_{j}^{m},T_{k}^{n}\right)$ as preliminarily processed.14:           **else if** $\left(T_{j}^{m},T_{k}^{n}\right)$ has been preliminarily processed **then**15:               Mark the pair $\left(T_{j}^{m},T_{k}^{n}\right)$ as complete processed.16:           **end if**17:        **end if**18:    **else if** the current $\left(T_{j}^{m},T_{k}^{n}\right)$ belongs to two existing $T_{i1}$ and $T_{i2}$ **then**19:        **if** $T_{i1}$ and $T_{i2}$ satisfy the Equation (5) and (7) **then**20:           Merge $T_{i1}$ and $T_{i2}$.21:        **else**22:           **if** $\left(T_{j}^{m},T_{k}^{n}\right)$ has been unprocessed **then**23:               Mark the pair $\left(T_{j}^{m},T_{k}^{n}\right)$ as preliminarily processed.24:           **else if** $\left(T_{j}^{m},T_{k}^{n}\right)$ has been preliminarily processed **then**25:               Mark the pair $\left(T_{j}^{m},T_{k}^{n}\right)$ as complete processed.26:           **end if**27:        **end if**28:    **end if**29:**end while**


## 4. System Framework and Markov Random Field Model for Data Association

**Figure 4:**
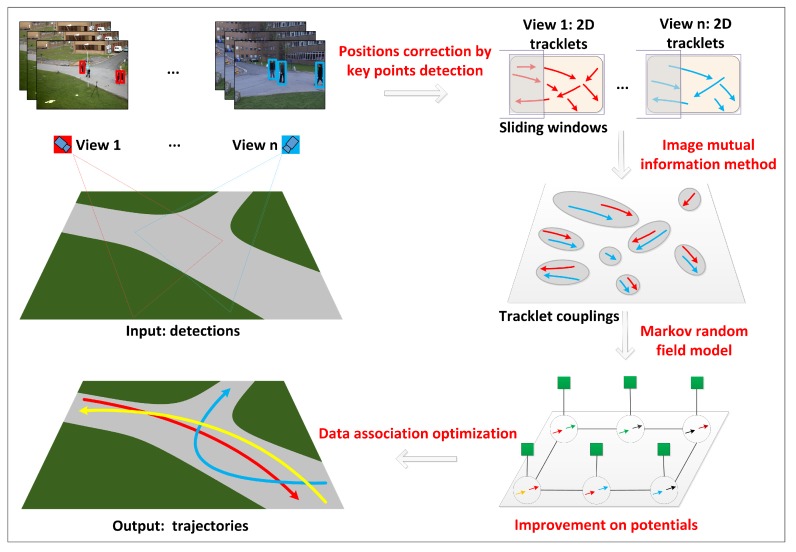
Framework of the proposed multi-view object tracking model, including position correction, tracklet building, cross-view tracklet coupling generation, the Markov random field model, potential function improvement, data association optimization, and the trajectories generation module.

The proposed multi-camera tracking system is presented in Figure 4. It consists of position correction, tracklet building, cross-view tracklet coupling generation, the Markov random field model, potential function improvement, data association optimization, and the trajectories generation.

The first three units in the framework have been discussed in the previous section and the tracklet coupling set $T=\{T_{i}\}$ was obtained. In the absence of severe occlusion, the length of the tracklet coupling is effectively expanded. However, in some cases of severe occlusions, especially in the case of dense crowds, a large number of tracklet couplings fragments are produced, making it impossible to obtain the complete trajectories of the objects. As the fragmented tracklet couplings contain scarce information, they cannot be connected well, even with the use of data association. For this purpose, we establish an MRF model in this study, and propose assimilation as well as extension processing and a message selection propagation algorithm (MSBP) to achieve the potential function improvement of fragmented tracklet couplings. This MSBP optimizes the MRF network parameters to obtain better trajectories of objects.

### 4.1. Markov Random Field Model

Among studies on MOT, the existing methods generally perform data association based on the correlations between the current tracklets. In the network flow model proposed in [8], each node represents a tracklet and the complete trajectories of objects are calculated by determining a global optimal association. The conditional random field model constructed in [32] considers the trajectory smoothness between nodes while searching for the optimal connection, while ensuring the reliability of the association. In [7], the mutual exclusion between node pairs was considered, on the basis of the work in [32], and the relationships between the difficulty pairs were handled more effectively. The hypergraph model constructed in [6] took into account the relationships among tracklets over a larger range and ensured reliable global association. When there are short and fragmented tracklets with scarce information, unreliable factors can be introduced into the data association, which reduces the overall accuracy of the association.

**Figure 5:**
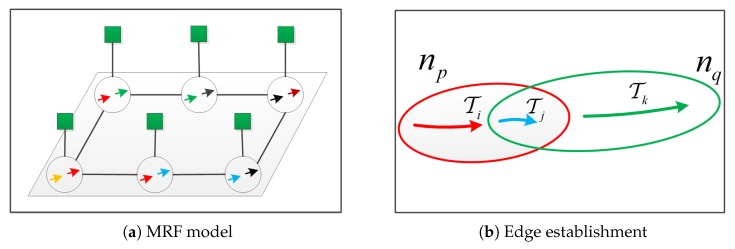
The proposed Markov random field model. In (**a**), the circle represents the node, while the block represents the observation information. The line between nodes is an edge; an edge holds only when two nodes contain the same tracklet coupling and the common tracklet coupling is in the middle position in the time relationship, as illustrated in (**b**).

In this paper, we establish an MRF to describe the association of T, as illustrated in Figure 5. The node $n_{p}\in N$, as represented by the circle in the figure, represents a link candidate for two tracklet couplings $T_{i}$ and $T_{j}$, where $T_{i}$ and $T_{j}$ satisfy the time-successive relationship and the time interval is less than the threshold of the discontinuous processing. Each node has a corresponding observation node $y_{m}\in Y$ which, as represented by the block in the figure, reflects the observation data of the corresponding tracklet couplings of the node. The edge $e_{pq}\in E$ between $n_{p}\left(T_{i},T_{j}\right)$ and $n_{q}\left(T_{j},T_{k}\right)$ is established on the condition that they contain the same $T_{j}$ and meets the requirement that $T_{i}$, $T_{j}$, and $T_{k}$ are successive in time. The state $l_{p}$ of node $n_{p}$ is binary, where $l_{p}=1$ represents that $T_{i}$ and $T_{j}$ in the node are in a connected state. Conversely, $l_{p}=0$ represents a disconnected state. The state of the nodes in the Markov network is implicit and the set of states of all nodes in the network is represented as $L=\{l_{1},...,l_{N}\}$. When a node contains only one $T_{k}$, this indicates that it is either a complete trajectory or a false alarm.

According to the research in [17], in an MRF containing a set $X=\{x_{p}\}$ of implicit nodes and a set of observation nodes $Y=\{y_{p}\}$, the joint posterior probability of the implicit nodes can be calculated by Equation (9), where $\psi_{p}\left(x_{p},y_{p}\right)$ is the local evidence of the node (i.e., the observation probability) and $\psi_{pq}\left(x_{p},x_{q}\right)$ is the compatible matrix of nodes $n_{p}$ and $n_{q}$:

(9) $$\begin{array}{c} P\left(X|Y\right)\propto \prod_{p}\psi_{p}\left(x_{p},y_{p}\right)\prod_{p}\prod_{q\in Ne(p)}\psi_{pq}\left(x_{p},x_{q}\right). \end{array}$$

In Equation (9), *X* and *Y* correspond to *L* and *Y*, respectively, in this model. We set

(10) $$\begin{array}{c} \psi_{p}\left(x_{p},y_{p}\right)=\psi_{p}\left(l_{p}\right)=\exp\left(-\phi \left(l_{p}\right)\right), \end{array}$$

(11) $$\begin{array}{c} \psi_{pq}\left(x_{p},x_{q}\right)=\psi_{pq}\left(l_{p},l_{q}\right)=\exp\left(-\varphi \left(l_{p},l_{q}\right)\right). \end{array}$$

In Equation (10), $\phi \left(l_{p}\right)=\phi \left(T_{i},T_{j}\right)$ is the observation of the similarity between the two tracklet couplings contained in node $n_{p}=\left(T_{i},T_{j}\right)$, where the appearance and motion information of $T_{i}$ and $T_{j}$ jointly determine $\phi (T_{i},T_{j})$ as shown in Equation (12):

(12) $$\begin{array}{c} \phi \left(T_{i},T_{j}\right)=\Lambda_{a}\left(T_{i},T_{j}\right)\Lambda_{m}\left(T_{i},T_{j}\right). \end{array}$$

Similar to the method in [14], the appearance similarity $\Lambda_{a}\left(T_{i},T_{j}\right)$ in this model is calculated based on the traditional appearance feature extraction method. The appearance similarity $\Lambda_{{}_{a}}^{v}\left(T_{i},T_{j}\right)$ between the two tracklet couplings in each view is calculated separately, and the multi-view overall appearance similarity $\Lambda_{{}_{a}}^{V}\left(T_{i},T_{j}\right)$ is determined jointly by Equation (13), $T_{s}^{v}\in T_{i}$ and $T_{t}^{v}\in T_{j}$, |V| is the number of views.

(13) $$\begin{array}{c} \Lambda_{{}_{a}}^{V}\left(T_{i},T_{j}\right)=\frac{1}{\left|V\right|}\sum_{v\in V}\sum_{s}\sum_{t}\Lambda_{{}_{a}}^{v}\left(T_{s}^{v},T_{t}^{v}\right). \end{array}$$

The motion similarity $\Lambda_{m}\left(T_{i},T_{j}\right)$ calculation method is similar to the method for single-view MOT [9,10,11]. It involves performing motion estimation of two tracklet couplings ${\tilde{T}}_{i}$ and ${\tilde{T}}_{j}$, calculating the distance $D({\tilde{T}}_{i},{\tilde{T}}_{j})$ between their estimated locations, and using Gaussian function for the motion similarity calculation, as shown in Equation (14):

(14) $$\begin{array}{c} \Lambda_{m}\left(T_{i},T_{j}\right)=G\left(D\left({\tilde{T}}_{i},{\tilde{T}}_{j}\right),0,\sigma \right). \end{array}$$

In Equation (15), $\varphi (l_{p},l_{q})$ is jointly determined by the motion and appearance relationships of the couplings contained in two nodes $n_{p}\left(T_{i},T_{j}\right)$ and $n_{q}\left(T_{j},T_{k}\right)$:

(15) $$\begin{array}{c} \varphi \left(l_{p},l_{q}\right)=\left\{\begin{array}{c} \Lambda (T_{i}+T_{j},T_{j}+T_{k})\\ \min[\Lambda \left(T_{i},T_{j}\right),\Lambda \left(T_{j},T_{k}\right)] \end{array}\right.\begin{array}{c} if\left|T_{j}\right|<\tau_{\varphi }\\ else, \end{array} \end{array}$$

where $\Lambda \left(T_{p},T_{q}\right)=\Lambda_{a}\left(T_{p},T_{q}\right)\Lambda_{m}\left(T_{p},T_{q}\right)$, $\left|T_{j}\right|$ is the frame length of the common coupling, and $\tau_{\varphi }$ is the frame length threshold. If the common coupling is short, the two nodes exhibit dependencies and the cascade similarity of the respective couplings is calculated. Otherwise, the similarity of the weaker is calculated.

Finally, the posterior probability of the MRF is transformed into the following.

(16) $$\begin{array}{c} P\left(L|T\right)\propto \prod_{p\in N}\exp(-\phi \left(l_{p}\right))\prod_{p}\prod_{q\in Ne(p)}\exp(-\varphi \left(l_{p},l_{q}\right)). \end{array}$$

### 4.2. Improvement of Potentials of Nodes Containing Small Tracklet Couplings

In multi-view MOT, dense crowd scenarios may cause frequent occlusions of the objects, often resulting in a large number of small tracking fragments. The appearance and motion characteristics of the small tracklet couplings formed by these short tracking fragments are not abundant or accurate, which affects the similarity calculation between tracklet couplings and leads to erroneous data association.

The majority of conventional schemes directly use the obtained network parameters to perform optimization after the establishment of the association model without specifically addressing the inaccuracy of the parameters. In this study, the nodes with short tracklet couplings are processed before data association is performed; furthermore, the related potential functions are improved to lay the foundation for subsequent reliable network solutions. In particular, this includes the following three steps.

**Figure 6:**
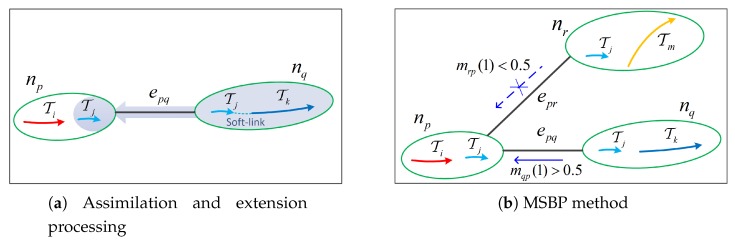
Diagram of assimilation, extension processing, and the MSBP method: (**a**) The soft link and assimilation in the right node, where the assimilation extends to the left node through $T_{j}$. Panel (**b**) demonstrates that the left node $n_{p}$ accepts the message passed by the right node $n_{q}$ and does not adopt the message passed by node $n_{r}$.

The first step is the assimilation and extension processing. In multi-view tracking of dense pedestrian crowds, the MRF model often contains both long tracklet couplings and a large number of fragmented tracklet couplings. As some local information is reliable and can be mined, we propose a method in this paper that starts from reliable tracklet couplings and then enhances the assimilation of adjacent small tracklet couplings and their extension processing. Beginning from the node of the longest tracklet coupling, if the similarity reaches the threshold, then they should be connected internally, which is called a soft link (for soft connections, real connection processing is not performed). After tracklet coupling in the soft-connect node is temporarily cascaded, the appearance and motion are recalculated, such that the two tracklet couplings are assimilated and the short tracklet coupling information is enhanced. Then, as a shared tracklet coupling, it directly affects the neighboring adjacent nodes, which is called assimilation extension processing, as illustrated in Figure 6. Assimilation and extension serve to improve the potential functions between the nodes.

The second step is MSBP processing. We use the belief propagation algorithm to calculate the marginal probability $P(l_{p})$ of each node state. Let $m_{p}\left(l_{p}\right)$ be the (normalized) local message sent by observation of node $n_{p}$, as indicated in Equation (17):

(17) $$\begin{array}{c} m_{p}\left(l_{p}\right)=\psi_{p}\left(l_{p}\right); \end{array}$$

$m_{pq}\left(l_{q}\right)$ is the (normalized) message sent to $n_{q}$ by node $n_{p}$, as indicated in Equation (18):

(18) $$\begin{array}{c} m_{pq}\left(l_{q}\right)\propto \sum_{p}\psi_{p}\left(l_{p}\right)\psi_{pq}\left(l_{p},l_{q}\right)\prod_{r\in Ne(p)\backslash q}m_{rp}\left(l_{p}\right). \end{array}$$

Unlike conventional processing [16,17], we introduce a special message selection process into the belief propagation algorithm. It can be seen, from the definition of Equation (15), that when a common tracklet coupling is short, the associated potential function of nodes $n_{p}$ and $n_{q}$ is calculated based on the internal cascade result of the two nodes. Based on this special background, we adopt a message selection rule for short common tracklet couplings, as follows.

(19) $$\begin{array}{c} m_{pq}\left(l_{q}\right)=\left\{\begin{array}{c} m_{pq}\left(l_{q}\right)\\ m_{pq}\left(0\right)=m_{pq}\left(1\right)=0.5 \end{array}\begin{array}{cc} & \begin{array}{c} if\;m_{pq}\left(1\right)>0.5\\ else. \end{array} \end{array}\right.\; \end{array}$$

It means that, if the message of node $n_{p}$ to node $n_{q}$ is biased toward a link, then $n_{q}$ accepts the message; otherwise, $n_{q}$ does not accept the message (i.e., the message is set to be a binary equal probability distribution). It can prevent the propagation of unreliable messages introduced by small tracklet couplings.

In the third step, the MSBP described above is iteratively performed until the marginal distribution of all nodes tends to stability or a termination condition is met. After the iteration procedure is complete, we verify the resulting marginal probabilities: $P(l_{p})$ is the probability of node $n_{p}$ is calculated by Equation (20).

(20) $$\begin{array}{c} P\left(l_{p}\right)\propto m_{p}\left(l_{p}\right)\prod_{q\in Ne(p)}m_{qp}\left(l_{p}\right). \end{array}$$

If the node contains a small tracklet coupling, we specify the following,

(21) $$\begin{array}{c} m_{p}\left(l_{p}\right)=\left\{\begin{array}{c} \max\{P\left(l_{p}\right),m_{p}\left(l_{p}\right)\}\\ m_{p}\left(l_{p}\right) \end{array}\begin{array}{cc} & \begin{array}{c} if\;P(l_{p})>0.5\\ else. \end{array} \end{array}\right.\; \end{array}$$

The local message is associated with the local potential function, and the potential function is thereby improved.

The above three processes are also performed in combination with the iterative strategy; that is, returning to the first step after the third step, searching for a reliable node that contains longer couplings, and performing processing again until the nodes whose tracklet coupling lengths are greater than the threshold have all fulfilled assimilation and extension, as well as the potential function improvement tasks. By improving the potential functions, the MRF’s parameters are optimized.

To generate trajectories of objects, in the existing research, based on the similarity between tracklet couplings, data association can be performed by the global dynamic programming algorithm [11], the continuous shortest path algorithm [10], or the minimum cost flow algorithm [8], as well as others. The global optimization-based methods can comprehensively make use of the relationship between trajectory segments and obtain the optimal association of multiple object trajectories from a global context. When the similarities exhibit high discrimination and accuracy, the local connection schemes, such as those presented in [7,37,38], can often achieve a global solution with better system efficiency. In the practical implementation, the MSBP method adopts maximum selection rule instead of Equation (19), i.e., only the maximum message is accepted. This simplifies the processing and still achieves good results. The stitching of complete trajectories is performed using the trajectory smoothness fitting method.

## 5. Experiment

In this section, we first introduce the evaluation indicators and experimental data sets. Then, we separately discuss the performance of key point optimization method, the tracklet couplings generation method, and the MRF data association optimization method. Finally, we compare the overall tracking system performance with that of other methods.

### 5.1. Evaluation Metrics and Experimental Dataset

Multi-view tracking performance can be evaluated using a single-view object-tracking evaluation system. In studies on multi-view tracking, one view is the main field of view and other views play an auxiliary role. Therefore, the authors of [13,14] proposed a feasible scheme using the tracking performance of the main view as the evaluation index for multi-view tracking. The main performance indicators include the tracking accuracy (MOTA), tracking precision (MOTP), mostly tracked target (MT), mostly lost target (ML), false negative (FN), false positive (FP), and object identity switches (IDs).

In this subsection, the whole tracking system is evaluated using the PETS2009 data set, where the evaluation metrics are those which were given in [41,42]. According to Equation (22), the MOTA combines $FP_{t}$, $FN_{t}$, and $IDs_{t}$ in frame *t*, and is given by

(22) $$\begin{array}{c} MOTA=1-\frac{\sum_{t}FN_{t}+FP_{t}+IDs_{t}}{\sum_{t}GT_{t}} \end{array}$$

$GT_{t}$ is the ground truth. The MOTP indicates the misalignment between tracked bounding boxes and their ground truth, and is given by

(23) $$\begin{array}{c} MOTP=\frac{\sum_{t}\sum_{i}D_{t}^{i}}{\sum_{t}M_{t}} \end{array}$$

where $M_{t}$ is the number of correct matches between target tracking results and GT in frame *t*, and $D_{t}^{i}$ is the distance of each match. The MT is the ratio of GT that are covered by a track hypothesis for at least 80% of their respective life span, whereas the ML is the ratio of GT that are covered by a track hypothesis for at most 20% of their respective life span. FP and FN are the total number of false positives and missed targets, respectively. IDs represent the total number of identity switches.

The PETS2009 data set [43] provides three experimental data sets for multi-view MOT with overlapping fields of view; namely, the S2.L1, S2.L2, and S2.L3 video sequences. The difficulty levels are L1, L2, and L3 from low to high, respectively, according to crowd intensity. At the same time, the image resolution of the three experimental sets is not very high; therefore, the appearance feature extraction and target 3D reconstruction accuracy exhibit large deviations, compared to the results obtained when using high-resolution images. These three experimental sequences are, thus, difficult and can be used as experimental sequences for evaluating tracking methods.

### 5.2. Evaluation of Key Point Optimization

**Figure 7:**
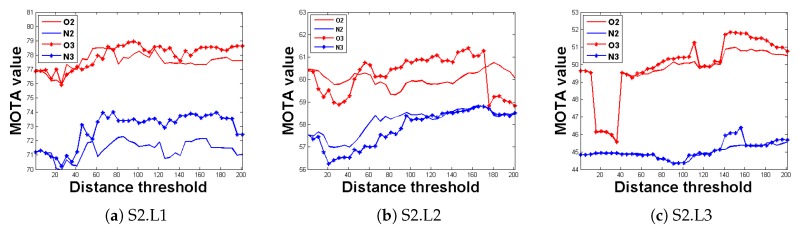
Evaluation results of the key point optimization. The horizontal axis represents the distance threshold, while the vertical axis represents the tracking accuracy. The solid line indicates that the experiment is conducted using data from two views, while the asterisk line indicates that the experiment is conducted using data from three views. Red represents the tracking system with key point optimization and blue indicates the tracking system without key point optimization.

In this subsection, the impact of the key point optimization on the whole tracking system is evaluated. Comparison experiments with and without key point optimization module were conducted using the three data sets S2.L1, S2.L2, and S2.L3 and the experimental results are presented in Figure 7.

The abscissa is the distance threshold between the tracklets, the vertical axis represents the tracking accuracy MOTA. The three charts represent the experimental results for S2.L1, S2.L2, and S2.L3, respectively. It can be seen the red curves are obviously better than the blue ones. This suggests that key point optimization is very helpful and improves the tracking performance largely.

### 5.3. Evaluation of Tracklet Coupling Generation

**Figure 8:**
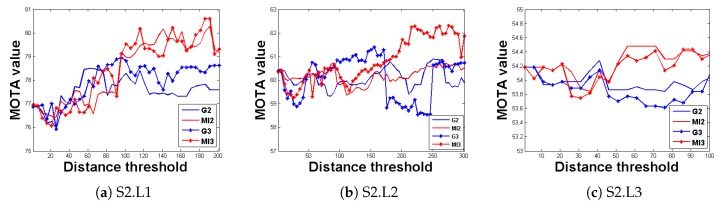
Evaluation results of tracklet coupling generation. The horizontal axis represents the distance threshold, whereas the vertical axis represents the tracking accuracy. The solid line indicates that the experiment is conducted using data from two views, while the asterisk line indicates that the experiment is conducted using data from three views. Blue represents the Gaussian distance metric and red represents the coupling method based on image mutual information.

In this subsection, we analyze and compare the coupling method based on a Gaussian distance metric and image mutual information. We separately replaced the coupled tracklet generation modules in the overall tracking framework and keep the other module parameters unchanged to observe the influence of different coupling methods on system tracking performance. The parameters of the Gaussian distance measurement method and the image mutual information method were set according to the current threshold $\delta_{i}$. The mean μ of the Gaussian distance measurement function was set to 0, $\sigma =2\delta_{i}/3$, and the Gaussian window size in the mutual information method was $s=3\delta_{i}/2$. Comparison experiments were conducted using the three data sets S2.L1, S2.L2, and S2.L3 and the experimental results are presented in Figure 8.

The abscissa is the distance threshold between the tracklets: when the minimum distance between two tracklets was greater than the threshold, the coupling operation was not performed. The vertical axis represents the tracking accuracy. The three curve charts represent the experimental comparison result curves for S2.L1, S2.L2, and S2.L3, respectively. The red curve represents the mutual information coupling method, the blue curve represents the Gaussian distance measurement method, the solid line represents the coupling method using data from two views, and the asterisk line represents the coupling method using data from three views. It can be seen, from the three curves, that when the distance threshold was 0 (i.e., no coupling operation was performed), an increasing number of tracklets participated in coupling as the distance threshold increased, and the tracking performance also improved. This suggests that the use of multiple view data for coupling helped the improve tracking performance. In Figure 8a,c, the mutual information method appeared to have equal or superior results to the Gaussian distance measurement method within the entire threshold range. In Figure 8b, although the tracking performance of the mutual information method did not exceed the conventional method locally, it was better within other threshold ranges and had a significant advantage; furthermore, its peak value was higher than that of the Gaussian distance measurement method. In summary, as indicated by the experimental results, the mutual information method is feasible and produces superior results.

### 5.4. Evaluation of Markov Random Field Optimization Method

In this subsection, we evaluate the data association optimization method based on MRF. Under the condition that the other module parameters of the system remained unchanged, the analysis was performed by comparing the impact on system performance with and without the optimization module. The final trajectory solutions all adopted the data association solving algorithm given in [38], to ensure the validity and fairness of the comparison. Experiments were conducted using the three data sets S2.L1, S2.L2, and S2.L3 and the experimental results are presented in Table 1. The direction of the arrow indicates good performance. The methods used data from three views to perform tracking. The difference was that, in data association, the normal method directly used the current trajectory similarity to generate the trajectory, whereas the MRF method optimized the tracklet similarity first and then generated the trajectory. The experimental results indicate that the MRF method is effective for optimizing the tracklets and the MOTA is improved through the optimization of data association. It is worth noting that during the optimization process some fragmented tracklets were activated, and that a decrease in the FN and IDs indicates the successful connection of difficult tracklet pairs.

**Figure 9:**
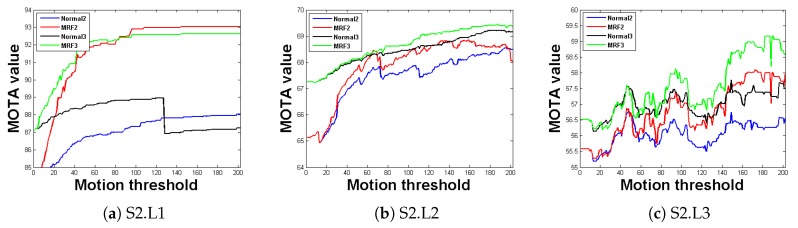
Evaluation results of MRF model. The horizontal axis represents the motion relationship threshold, while the vertical axis represents the tracking accuracy. The black curve represents the potential function improvement method with three views, the green curve represents the conventional method with three views, the blue curve represents the potential function improvement method with two views, and the red curve represents the conventional method with two views.

To provide an effective analysis and discussion, the other parameters were kept unchanged. By changing the motion similarity threshold, the system tracking performance could be observed, as illustrated in Figure 9. Four experiments were conducted for each experimental sequence; that is, for the conventional methods (red) with two views, the MRF method (blue) with two views, the conventional method (green) with three views, and the MRF method with three views. Figure 9 demonstrates that, in the three sequences, the MRF method maintained an advantage in tracking performance over the conventional method with an increase in the motion similarity threshold. In addition, regardless of the method used, the tracking performance of the tracking system with three views was superior to that of the tracking system with two views, which also suggests that the tracking system in this study functions properly.

## 6. Discussion and Conclusions

In this paper, we used a multi-camera system with overlapping fields of view to study the problem of dense pedestrian tracking and proposed a new MRF model for cross-view tracklet couplings. This model is equipped with a new potential function improvement method that can perform effective association of tracklet coupling fragments caused by dense crowds. To generate reliable tracklet couplings, a data fusion method based on image mutual information was proposed. This method can calculate the spatial relationships of cross-view 2D tracklet pairs by integrating position and motion information. The human key point detection method was also adopted to correct the position data of incomplete and deviated objects in dense crowds.

We made use of the PETS2009 experimental data set for modular experiment. From the experimental results, human key points can effectively improve the object detection in dense pedestrians scene and lead to better 3D reconstruction of tracklet. The data fusion method based on image mutual information combines the motion and position information of the tracklets and provides a more discriminative spatial relationship. The potential function improvement method of our MRF model helps association of fragmented tracklet couplings in the case of dense crowds. These three steps provide an effective solution to the occlusion problem in dense pedestrians tracking.

We also provide comparisons between the tracking system proposed in this study and existing methods, such as those presented in [12,13,14,28]. The comparison results are presented in Table 2. The input and output evaluations of the experimental results were consistent with the evaluation system in [14], and the same input and ground truth were used to ensure the fairness of the comparison.

**Figure 10:**
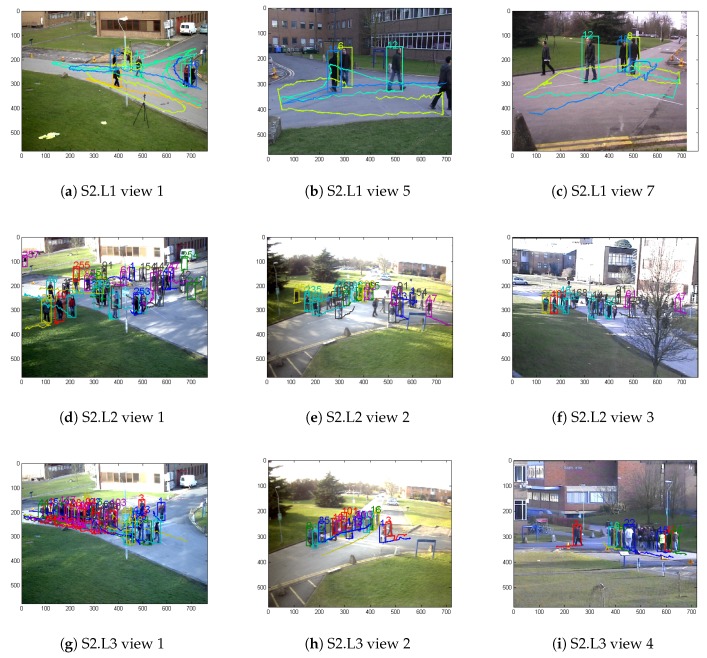
Tracking results. Panels (**a**–**c**) display the tracking results of the first to the 350th frame of the first, fifth, and seventh views in PETS2009 S2.L1, respectively. Panels (**d**–**f**) display the tracking results of the first to the 51st frame of the first, second, and third views in PETS2009 S2.L2, respectively. Panels (**g**–**i**) display the tracking results of the 160th to the 210th frame of the first, second, and fourth views in PETS2009 S2.L3, respectively.

Table 2 indicates that the tracking system performance in this study achieved favorable results with the S2.L1 experimental sequence, although it did not exceed the method of [14], the 100% MT and lower IDs values surpassed the other methods. In the more difficult dense crowd scenes of S2.L2 and S2.L3, our method achieved the best results, indicating that the Markov optimization model plays a key role in processing dense scenarios. The table also indicates that the method in this study makes better use of multi-view data information for tracking. In the three experimental sequences, the tracking performance using three views was superior to the tracking performance using only two views, which is consistent with the actual physical meaning and the original intention of multi-view research. Figure 10 presents the partial tracking results of the three experimental sequences, indicating that the object trajectory can be estimated more accurately when tracking is performed using the information of multiple views. At the same time, it can also be seen that the same object was correctly assigned the same tracking number in different views.

In future research, cross-view feature extraction will be a core task. Due to difference in views between cameras, backgrounds can be quite different and the same target may have a different appearance. The direct use of traditional feature extraction methods may not be able to effectively identify the same object or distinguish among different objects. In recent years, the development of deep learning in the field of pedestrian recognition has supplied new solutions for cross-view appearance feature extraction. Consequently, deep-learning-based cross-view appearance feature extraction will be the next major focus of our research.

## Figures and Tables

**Table 1:** Results of comparison between the MRF optimization method and a conventional method.

Sequences	Methods	MOTA↑	MOTP↑	GT	MT↑	PT↑	ML↓	FP↓	FN↓	IDs↓
S2.L1	Normal	88.04	76.58	19	19	0	0	138	363	55
	MRF	93.03	76.59	19	19	0	0	144	173	7
S2.L2	Normal	70.38	72.86	43	30	13	0	832	1869	347
	MRF	70.64	72.84	43	30	13	0	844	1844	337
S2.L3	Normal	57.79	70.81	44	22	19	3	398	1248	201
	MRF	58.98	70.75	44	23	18	3	429	1182	184

**Table 2:** Comparisons of tracking performance.

Sequences	Methods	Camera ID	MOTA↑(%)	MOTP↑(%)	GT	MT↑(%)	ML↓(%)	IDs↓
S2.L1	Method1 [12]	1, 5	85.74	67.87	19	89.47	0.00	150
		1, 5, 7	82.06	66.23	19	89.47	0.00	270
	Method2 [13]	1, 5	91.89	79.50	19	94.74	0.00	41
		1, 5, 7	91.66	79.40	19	94.74	0.00	45
	Method3 [14]	1, 5	95.51	80.60	19	100.00	0.00	14
		1, 5, 7	95.08	79.80	19	100.00	0.00	13
	Method4 [28]	1, 3	76.33	65.28	19	92.59	0.71	2
	Proposed	1, 5	92.62	76.49	19	100.00	0.00	10
		1, 5, 7	93.03	76.59	19	100.00	0.00	7
S2.L2	Method1 [12]	1, 2	40.14	54.13	43	4.62	9.30	621
		1, 2, 3	36.38	53.83	43	2.33	9.30	865
	Method2 [13]	1, 2	58.97	65.80	43	25.56	2.33	385
		1, 2, 3	58.85	66.00	43	30.23	2.33	388
	Method3 [14]	1, 2	67.00	61.50	43	51.16	0.00	239
		1, 2, 3	65.24	61.80	43	44.19	0.00	249
	Proposed	1, 2	69.41	72.83	43	65.12	0.00	288
		1, 2, 3	70.11	72.84	43	69.77	0.00	337
S2.L3	Method1 [12]	1, 2	48.49	51.74	44	22.73	9.09	279
		1, 2, 4	40.55	49.46	44	9.09	15.71	300
	Method2 [13]	1, 2	54.39	60.20	44	25.00	25.00	106
		1, 2, 4	49.79	63.00	44	29.55	25.00	123
	Method3 [14]	1, 2	57.06	59.30	44	38.64	15.91	129
		1, 2, 4	54.39	54.90	44	29.55	20.45	92
	Proposed	1, 2	58.00	71.01	44	50.00	9.09	188
		1, 2, 4	59.16	70.82	44	52.27	6.82	167

## Appendix A

Suppose an image of size $N_{0}$ has $n_{i}$ pixels of value *i*, where 0≤i≤255. The information entropy is calculated as follows,

(A1) $$\begin{array}{c} \begin{array}{c} H_{0}\left(x\right)=-\sum_{i=0}^{255}P(x=i)\log P(x=i)\\ =-\sum_{i=0}^{255}\frac{n_{i}}{N_{0}}\log\frac{n_{i}}{N_{0}}\\ =-\frac{1}{N_{0}}\sum_{i=0}^{255}n_{i}\left(\log n_{i}-\log N_{0}\right)\\ =-\frac{1}{N_{0}}\left(\sum_{i=0}^{255}n_{i}\log n_{i}+\log N_{0}\sum_{i=0}^{255}n_{i}\right)\\ =-\frac{1}{N_{0}}\sum_{i=0}^{255}n_{i}\log n_{i}+\log N_{0} \end{array} \end{array}$$

Suppose that the image is expanded to size of $N_{1}=N_{0}+N_{a}$ by adding $N_{a}$ zero value pixels. Noting that the new image has $n_{0}^{\prime }=n_{0}+N_{a}$ pixels of zero value, its information entropy $H_{1}\left(x\right)$ satisfies Equation (25), that is,

(A2) $$\begin{array}{c} \begin{array}{c} H_{1}\left(x\right)=-\frac{1}{N_{1}}\left({n^{\prime }}_{0}\log{n^{\prime }}_{0}+\sum_{i=1}^{255}n_{i}\log n_{i}\right)+\log N_{1}\\ =-\frac{{n^{\prime }}_{0}\log{n^{\prime }}_{0}-n_{0}\log n_{0}}{N_{1}}-\frac{1}{N_{1}}\sum_{i=0}^{255}n_{i}\log n_{i}+\log N_{1}\\ =-\frac{{n^{\prime }}_{0}\log{n^{\prime }}_{0}-n_{0}\log n_{0}}{N_{1}}+\frac{N_{0}}{N_{1}}\left(-\frac{1}{N_{0}}\sum_{i=0}^{255}n_{i}\log n_{i}+\log N_{0}\right)-\frac{N_{0}}{N_{1}}\log N_{0}+\log N_{1}\\ =\frac{N_{0}}{N_{1}}H_{0}\left(x\right)-\frac{{n^{\prime }}_{0}\log{n^{\prime }}_{0}-n_{0}\log n_{0}}{N_{1}}-\frac{N_{0}}{N_{1}}\log N_{0}+\log N_{1}\\ =\beta H_{0}\left(x\right)+f\left(n_{0},N_{0},N_{a}\right) \end{array} \end{array}$$

where, $\beta =\frac{N_{0}}{N_{0}+N_{a}}$ and $f\left(n_{0},N_{0},N_{a}\right)=-\frac{\left(n_{0}+N_{a}\right)\log\left(n_{0}+N_{a}\right)-n_{0}\log n_{0}}{N_{0}+N_{a}}-\frac{N_{0}}{N_{0}+N_{a}}\log N_{0}+\log\left(N_{0}+N_{a}\right)$.
